# Magnetosomes for bioassays by merging fluorescent liposomes and magnetic nanoparticles: encapsulation and bilayer insertion strategies

**DOI:** 10.1007/s00216-020-02503-0

**Published:** 2020-02-18

**Authors:** Cornelia A. Hermann, Carola Hofmann, Axel Duerkop, Antje J. Baeumner

**Affiliations:** Institute of Analytical Chemistry, Chemo- and Biosensors, University of Regensburg, Universitätsstraße 31, 93053 Regensburg, Germany

**Keywords:** Magnetic nanoparticles, Fluorescent liposomes, DNA sandwich hybridization assay, Magnetosomes, Signal enhancement

## Abstract

Magnetized liposome (magnetosomes) labels can overcome diffusion limitations in bioassays through fast and easy magnetic attraction. Our aim therefore was to advance the understanding of factors influencing their synthesis focusing on encapsulation strategies and synthesis parameters. Magnetosome synthesis is governed by the surface chemistry and the size of the magnetic nanoparticles used. We therefore studied the two possible magnetic labelling strategies, which are the incorporation of small, hydrophobic magnetic nanoparticles (MNPs) into the bilayer core (b-liposomes) and the entrapment of larger hydrophilic MNPs into the liposomes’ inner cavity (i-liposomes). Furthermore, they were optimized and compared for application in a DNA bioassay. The major obstacles observed for each of these strategies were on the one hand the need for highly concentrated hydrophilic MNPs, which is limited by their colloidal stability and costs, and on the other hand the balancing of magnetic strength vs. size for the hydrophobic MNPs. In the end, both strategies yielded magnetosomes with good performance, which improved the limit of detection of a non-magnetic DNA hybridization assay by a factor of 3–8-fold. Here, i-liposomes with a magnetization yield of 5% could be further improved through a simple magnetic pre-concentration step and provided in the end an 8-fold improvement of the limit of detection compared with non-magnetic conditions. In the case of b-liposomes, Janus-like particles were generated during the synthesis and yielded a fraction of 15% magnetosomes directly. Surprisingly, further magnetic pre-concentration did not improve their bioassay performance. It is thus assumed that magnetosomes pull normal liposomes through the magnetic field towards the surface and the presence of more magnetosomes is not needed. The overall stability of magnetosomes during storage and magnetic action, their superior bioassay performance, and their adaptability towards size and surface chemistry of MNPs makes them highly valuable signal enhancers in bioanalysis and potential tools for bioseparations.

**Electronic supplementary material:**

The online version of this article (10.1007/s00216-020-02503-0) contains supplementary material, which is available to authorized users.

**Figure:**
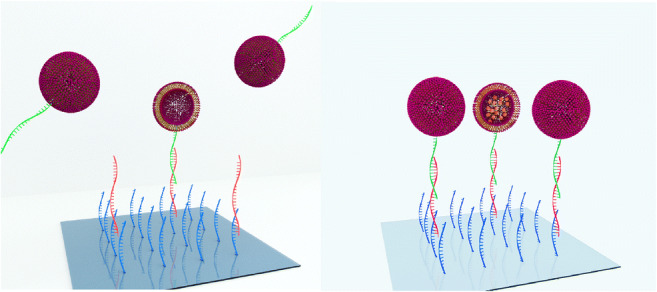
Graphical abstract

## Introduction

*Cryptosporidium parvum* is an intestinal parasite, which causes diarrhea amongst a wide range of human communities. At the moment, there only exists one proven anti-parasitic drug for cryptosporidiosis, but even this treatment is not effective for all groups of patients and especially infants and immunocompromised persons are still threatened by this pathogen [1–3]. This makes clear that a high necessity exists to detect this threat early, especially as it can even occur in treated drinking water [1].

A common technique for the recognition of pathogens is to detect their genome by DNA hybridization. Amongst these hybridization assays, sandwich assays hold the advantage of twofold recognition by one capture and one reporter probe, which yields higher selectivity. DNA sandwich hybridization assays have been reported for the detection of many pathogens, e.g., *Salmonella* [4], *hepatitis B* [5], *Bacillus anthracis* [6], and also *C. parvum* [7]. But this assay format heavily relies on diffusion and accidental meeting of the reaction partners in solution or, even worse, on diffusion to the surface of a sensor platform or microtiter plate. By overcoming these diffusion-based processes, faster and more efficient binding of the reaction partners would be possible, resulting in the possibility to apply these assays even more efficiently in sensors to achieve higher field portability and point of care devices, e.g., to detect low numbers of *C. parvum* in contaminated drinking water and food or in infected patients. One possible strategy for the overcoming of diffusion barriers is the directed attraction in an external electromagnetic field as established in many bioanalytical approaches taking advantage of immunomagnetic separation strategies [8–10]. Thus, bioanalytical labels should be modified to have a dual functionality: signal generation as well as magnetic attraction.

**Fig. 1:**
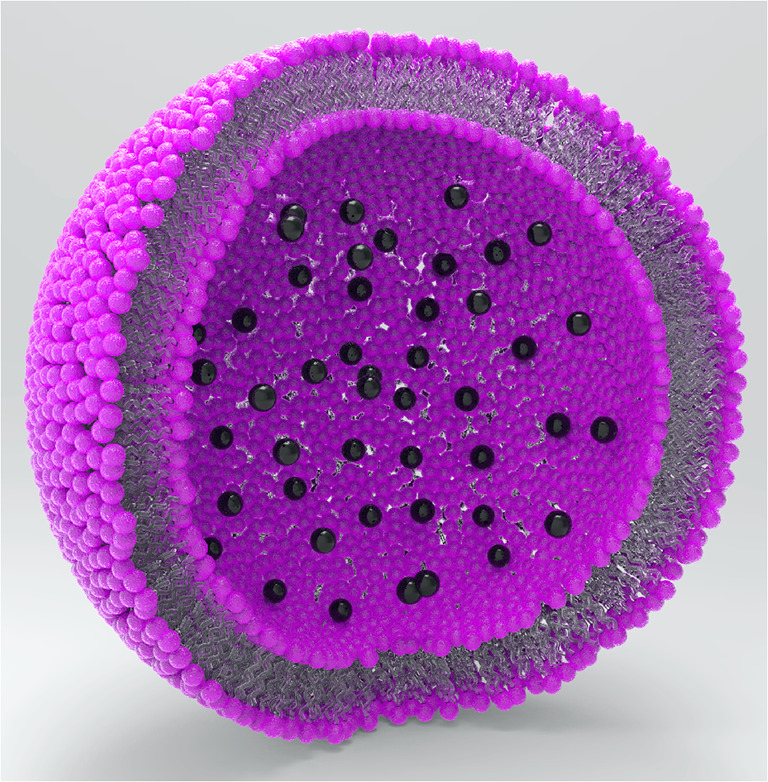
Partly scale drawing of a liposome (200 nm in diameter) with nanoparticles (10 nm in diameter) encapsulated into the hydrophilic lumen inside the liposome. Note: The bilayer thickness of the liposomes is *not* to scale, only the proportion of particles to liposomes

Liposomes—artificial vesicles filled with signal molecules—are applied often as bioanalytical labels due to their high biocompatibility and the attribute of signal amplification due to the release of a high number of dye molecules by one bound liposome [11]. By incorporation of magnetic material into a liposome, a so-called magnetosome, a liposome with magnetic properties, is formed. As magnetic material for incorporation, magnetic nanoparticles (MNPs) can be used, e.g., iron oxide nanoparticles. For the synthesis of magnetosomes, the reverse phase evaporation [7] as well as the thin film rehydration method [12] was employed. Both display the efficient encapsulation of molecules and yield long-term stable liposomes, and therefore were promising for the successful encapsulation of MNPs. With both methods, it is possible to either encapsulate MNPs in the hydrophilic inner volume together with signal molecules or separate from them in the hydrophobic lipid bilayer core. For the encapsulation into the hydrophilic inner volume, shown in Fig. 1, surface modification of MNPs is necessary as after synthesis they are coordinated by hydrophobic oleic acid. These modified nanoparticles can then be added together with the signal molecules as aqueous encapsulation solution.

**Fig. 2:**
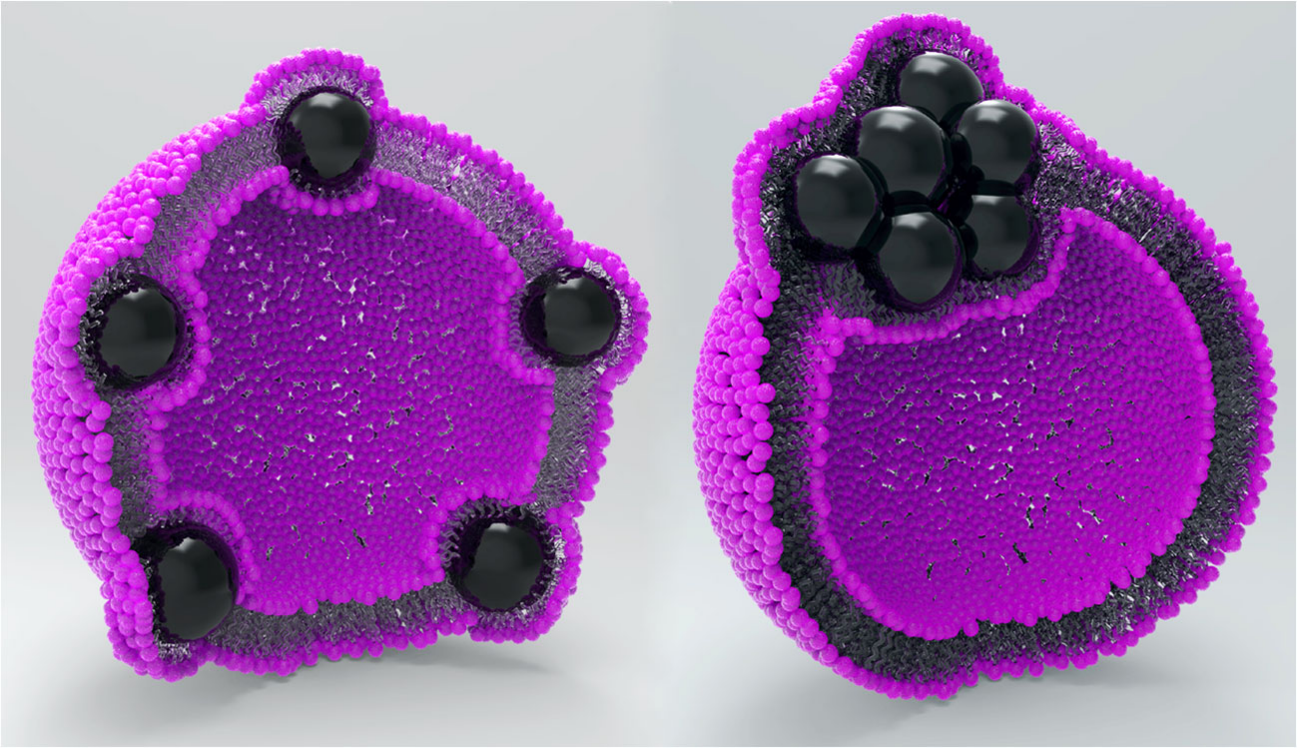
Partly scale drawing of liposomes with nanoparticles (10 nm in diameter) incorporated into the lipid bilayer core (4 nm in thickness). *Left*: This ideal scheme shows a statistical distribution of particles and the vast distortion of the membrane by the particles. *Right*: The more likely to be found liposome form will equal this Janus-shaped liposome with agglomerated MNPs on one side and the hydrophilic department on the other. Note: The diameter of liposomes is *not* to scale, only the proportion of particles to bilayer thickness

Alternatively, the as-synthesized hydrophobic MNPs can be incorporated into the lipid bilayer by simply adding them to the lipids dissolved in organic solvents. The magnetization power of MNPs scales with their size [13], but at the same time, they must fit into the ~ 4-nm lipid bilayer of the liposomes. While some studies and theoretical models show that the maximum particle diameter for insertion into the bilayer is about 5 nm [14], the successful insertion of particles with up to 15 nm diameter has been reported [15]. Figure 2 left shows an ideal scheme of this incorporation, where the particles are statistically distributed across the membrane. As the membrane has only about 4 nm in thickness and the particles have a diameter of around 10 nm, the membrane has to wrap around the particles, being distorted to a heavy degree. For this reason, the particles will most likely tend to agglomerate at one point and form kind of Janus-shaped vesicles [16], as shown in Fig. 2 right. As reporter molecules are assumed to be distributed across the membrane of the vesicles, this asymmetry is not suspected to influence the binding efficiency.

**Fig. 3:**
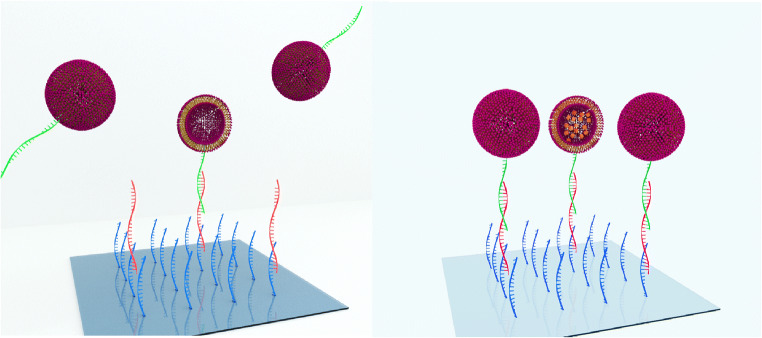
Comparison of a DNA hybridization assay with liposomes used for signal enhancement without (*left*) and with (*right*) MNPs incorporated into the liposomes under the influence of an external magnetic field. By magnetic attraction, more liposomes can bind to the target DNA

To use magnetosomes as analytical tool, their surface must be modified enabling coupling to bioreceptors. Appropriately modified lipids can be inserted directly during synthesis, or they can be inserted after synthesis by intercalation of the hydrophobic chains into the outer lipid bilayer [17, 18], or covalent coupling reactions can be performed with purified liposomes. Due to the simplicity and high reliability of the approach, insertion of cholesterol-tagged DNA reporter was chosen. For quality control of the resulting magnetosomes, their performance in bioassays in the presence or absence of an external electromagnetic field was compared. Sulforhodamine B was used as signal-generating molecule, a fluorescent dye that in high concentrations is self-quenching, ensuring that while encapsulated in liposomes, the dye is not detectable. By lysis of the liposomes, the dye is set free and diluted, which disables self-quenching of the fluorescence. Therefore, by a very small amount of bound liposomes, a clearly measurable signal can be generated [11]. With this assay, the ability of magnetosomes to improve a DNA sandwich hybridization assay for *C. parvum* was investigated. The magnetosomes cannot only be transported to their point of destination by diffusion but also directed by a magnetic field, and a greater amount of magnetosomes should be able to bind in shorter time than in a conventional diffusion-based assay, as shown in Fig. 3. By removal of the magnetic field and convection, the unspecifically bound magnetosomes should be detached and the signal-to-noise ratio may be considerably improved [15].

This work carefully examines and optimizes the different strategies to encapsulate MNPs into liposomes and compares their efficiency and the performance of the resulting magnetosomes in DNA sandwich hybridization assays with and without magnetic purification.

## Materials and methods

### Materials

Iron (III) chloride hexahydrate (≥ 99%) and sodium oleate (≥ 82%) were bought from Sigma-Aldrich (www.sigmaaldrich.com). Oleic acid and 1-octadecene (both 90%, technical grade) were obtained from Alfa Aesar (www.alfa.com). Iron (II, III) oxide magnetic nanoparticle solutions (30 nm average diameter, amine functionalized, 1 mg mL^−1^ in H_2_O) were obtained from Sigma-Aldrich (www.sigmaaldrich.com).

1,2-Dipalmitoyl-sn-glycero-3-phosphocholine (DPPC), 1,2-dipalmitoyl-sn-glycero-3-phospho-(1′-rac-glycerol) (sodium salt) (DPPG), 1,2-dipalmitoyl-sn-glycero-3-phosphoethanolamine-N-(biotinyl) (sodium salt) (biotin-DPPE), and 1,2-dipalmitoyl-sn-glycero-3-phosphoethanolamine-N-(glutaryl) (sodium salt) (N-glutaryl-DPPE) were purchased from Avanti Polar Lipids, Inc. (www.avantilipids.com); cholesterol and sulforhodamine B (SRB) were obtained from Sigma-Aldrich (www.sigmaaldrich.com); and n-octyl-β-D-glucopyranoside (OG) was bought from Roth (www.carlroth.com).

A DNA derived from the *C. parvum* heat shock protein 70 (hsp70) mRNA was used as model analyte for DNA hybridization assays. Three different sequences were employed, specified as capture probe (CP), target sequence (tDNA), and reporter probe (RP) (CP: 5′-biotinyl-AGA TTC GAA GAA CTC TGC GC-3′; tDNA: 5′-AAGGACCAGCATCCTTGAGTA CTTTCT C AA CTG GAG CTA AAG TTG CAC GGA AGT AAT CA GCG CAG AGT TCT TCG AAT CT AG CTC TAC TGA TGG CAA CTG A-3′; RP: 5′-GTG CAA CT T TAG CTC CAG TT-cholesteryl-3′). This DNA was obtained from Metabion (www.metabion.com).

Chloroform, cyclohexane, and methanol were purchased from Fisher Scientific (www.fishersci.com).

4-(2-Hydroxyethyl)-1-piperazineethanesulfonic acid (HEPES) and sodium azide were bought from Sigma-Aldrich (www.sigmaaldrich.com). BSA (albumin fraction V from bovine serum), di-potassium hydrogen phosphate trihydrate, di-sodium hydrogen phosphate dihydrate, formamide, potassium chloride, potassium dihydrogen phosphate, and tri-sodium citrate dihydrate were bought from Merck (www.merckmillipore.com). Ficoll 400 and sodium chloride were obtained from Roth (www.carlroth.com) and sucrose was purchased from VWR (de.vwr.com).

All other chemicals were of analytical grade and obtained either from VWR (de.vwr.com), Merck (www.merckmillipore.com), Roth (www.carlroth.com), or Sigma-Aldrich (www.sigmaaldrich.com). Double distilled water was used for the preparation of all aqueous solutions.

HEPES buffer consisted of 10 mM HEPES, 200 mM NaCl, and 0.01% (w/v) NaN_3_, and pH was adjusted to 7.5. For HSS buffer (HEPES-saline-sucrose), a varying amount of sucrose was added to adjust right osmolality for liposome outer buffer. Phosphate-buffered saline (PBS) contained 137 mM NaCl, 2.7 mM KCl, 10 mM Na_2_HPO_4_, and 1.8 mM KH_2_PO_4_ at pH 7.4. For production of washing buffer, 0.05% (v/v) Tween 20 and 0.01% (w/w) bovine serum albumin were added to PBS. Potassium phosphate buffer consisted of 50 mM K_2_HPO_4_, 50 mM KH_2_PO_4_, and 1 mM EDTA, and pH was adjusted to 7.5. Hybridization buffer was prepared from 1.35 M NaCl, 0.135 M sodium citrate, 0.01% (w/v) NaN_3_, 30% (v/v) formamide, and 0.2% (w/v) Ficoll 400 with pH 7.0.

For extrusion of liposomes, an extruder equipped with syringes, filter supports, and membranes from Avanti Polar Lipids, Inc. (www.avantilipids.com) was employed. Sephadex G50 for column chromatography was purchased from Sigma-Aldrich (www.sigmaaldrich.com).

Black MaxiSorp 96-well microtiter plates (MTPs) from Nunc for stability testing and magnetic washing experiments were purchased from Sigma-Aldrich (www.sigmaaldrich.com). For DNA hybridization assays, white streptavidin-coated MTPs (KaiSA 96) with a biotin-binding capacity of > 14 pmol/well from Kaivogen Oy (kaivogen.com) were used.

### Synthesis and characterization of magnetic nanoparticles

MNPs were synthesized by thermal decomposition, adapting a method developed by Park et al. [19], where an Fe(oleate)_3_ precursor is decomposed at high temperatures.

For the precursor synthesis, typically 2.703 g FeCl_3_·6H_2_O (1 equiv., 10 mmol) and 9.133 g sodium oleate (3 equiv., 30 mmol) are dissolved in a solvent mixture of 20 mL ethanol, 15 mL water, and 35 mL hexane and heated to reflux for 4 h. After cooling to room temperature, the organic phase was washed with 10 mL water three times and the solvent was removed at the rotary evaporator at reduced pressure.

For particle synthesis, typically 0.9 g precursor (1 equiv., 1 mmol) and 120 mg oleic acid (0.4 equiv., 0.4 mmol) were dissolved in 10 mL octadecene in a three-necked round-bottom flask. The flask is heated to 120 °C, flushed with nitrogen for 15 min, and then set under vacuum for another 15 min. Again nitrogen is applied and the solution is heated to reflux (> 320 °C) for 1 h. After cooling to room temperature rapidly, the nanoparticles are washed with cyclohexane and ethanol via centrifugation (3 times, 5 min, 4000*g*) and aggregates are removed by centrifugation for 3 min at 1000*g*. Afterwards, the nanoparticles are stored at 4 °C as dispersion in cyclohexane.

The method of choice for determination of diameter and uniformity of the particles was transmission electron microscopy (TEM). Therefore, a 120-kV Philips CM12 (ww.fei.com) microscope was employed and the obtained images were evaluated with ImageJ software (http://rsbweb.nih.gov/ij/). Liposome samples were stained with phosphotungstic acid for TEM. The hydro- and solvodynamic diameters (with polydispersity index (PdI)) were determined by dynamic light scattering (DLS) at 20 °C with a Malvern Zetasizer Nano-ZS (www.malvern.com) in disposable poly(methyl methacrylate) (PMMA) cuvettes (semi-micro). ζ-Potential measurements were carried out at the same instrument in disposable capillary cells. Hydro-/solvodynamic diameters were determined 13 times for each sample and averaged. The zeta potential was measured 25 to 37 times and averaged.

### Liposome synthesis and characterization

Liposomes were synthesized either by reverse phase evaporation according to an established procedure from Edwards et al. or by thin film rehydration according to a modified procedure by Bangham et al. [12]. Typically, 10 mg cholesterol (26 μmol, 46% of lipid composition), 15 mg DPPC (20 μmol, 36%), and 7.5 mg DPPG (10 μmol, 18%) are dissolved in 0.5 mL methanol and 3 mL chloroform. The solution is sonicated (VWR USC 300 THD/HF bath sonicator at maximum power, de.vwr.com) for 1 min.

For reverse phase evaporation, 2 mL encapsulant (10 mM SRB in 20 mM HEPES, pH 7.5) is added and the mixture is sonicated for further 4 min. Organic solvents are evaporated at the rotary evaporator at 60 °C under reduced pressure. The mixture is vortexed thoroughly and 2 mL encapsulant is added. The flask is transferred back to the rotary evaporator to get rid of any remaining organic solvent.

For thin film rehydration, first, organic solvents are evaporated at the rotary evaporator at 60 °C under reduced pressure. Then, 4 mL encapsulant is added for rehydration of the lipid film by rotating at atmospheric pressure at 60 °C at the rotary evaporator for 1 h. After 30 min, the liposome solution was vortexed thoroughly.

In both cases, the liposomes are extruded each 21 times at 60 °C through two polycarbonate membranes with 1.0 and 0.4 μm pores, respectively. The solution is purified by size exclusion column chromatography (1.5 × 20 cm, Sephadex G50) to remove free dye and free lipids. Liposome-containing fractions are collected as medium and high concentrated solutions and transferred to dialysis (pore size 12–14 kD, spectrumlab.com) 2 times for each 12 h against 600 mL HEPES buffer.

To achieve modified liposomes, different additions are possible: Reporter probe-modified liposomes are achieved by addition of 25 μL of a 300 μM solution of DNA tagged with cholesterol (15 nmol) with the lipid ingredients. For incorporation of hydrophobic-coated 8-nm MNPs into the lipid bilayer, 2.5 mg MNPs dispersed in cyclohexane are transferred into 1 mL chloroform by precipitation with ethanol, centrifugation, and redispersion in chloroform. This dispersion replaces 1 of 3 mL chloroform as lipid solvent. For encapsulation of MNPs with hydrophilic surface coating, 1 mL of the 1 mg mL^−1^ aqueous 30 nm MNP solution is added to the encapsulation solution and replaces 1 mL H_2_O for the encapsulant preparation.

Concentrations of phospholipids are determined by ICP-OES measurements of phosphor at either 177.495 nm or 213.618 nm on a Spectroflame-EOP inductively coupled plasma optical emission spectrometer (ICP-OES) from Spectro (www.spectro.com), hydrodynamic diameters by DLS at 20 °C with a Malvern Zetasizer Nano-ZS (www.malvern.com) in disposable PMMA cuvettes (semi-micro). ζ-Potential measurements were carried out at the same instrument in disposable capillary cells. Hydrodynamic diameters were determined 12 to 15 times for each sample and averaged, zeta potential 25 to 37 times.

### DNA hybridization assay

Streptavidin-coated microtiter plates were used for the following assay. All steps are performed in parallel on two identical MTPs.

Each well is washed with washing buffer (2 × 200 μL/well) and PBS (1 × 200 μL/well) and biotinylated capture probe (*C. parvum*, 0.1 μM in potassium phosphate buffer, 100 μL/well) is added. MTPs are incubated for 30 min at 23 °C and 300 rpm on an Eppendorf ThermoMixer C (online-shop.eppendorf.de). Unbound capture probe is removed and the wells are washed with washing buffer (2 × 200 μL/well) and hybridization buffer (1 × 200 μL/well).

Synthetic target DNA (*C. parvum*, different concentrations in hybridization buffer, 100 μL/well) is added and MTPs are incubated for 30 min at 23 °C on Eppendorf ThermoMixer C. Unbound target DNA is removed and the wells are washed with HEPES buffer (2 × 200 μL/well).

Liposome-reporter DNA (50 μM total lipid in HEPES buffer, 100 μL/well) is added and incubated for 60 min at room temperature, where only one MTP is placed on a permanent magnet. During this time, both MTPs are transferred two times to the ThermoMixer C and shaken for 10 s. Unbound liposomes are removed and the wells are washed with HEPES buffer (2 × 200 μL/well).

Fluorescence is measured in 100 μL HEPES buffer once either with a BioTek SYNERGY neo2 (www.biotek.com) or a FLUOstar® OPTIMA microtiter plate reader from BMG LABTECH (www.bmglabtech.com) at 544 nm excitation and 575 nm emission wavelength. Then, the supernatant is removed and octyl glycoside (30 mM, 100 μL/well) is added to induce lysis of the liposome bilayer. After 5-min incubation, fluorescence is measured again.

## Results and discussion

### Characterization of magnetic nanoparticles and liposomes

Iron oxide magnetic nanoparticles were synthesized using previously established protocols [19] and characterized by DLS and TEM. With DLS, a solvodynamic diameter of 17.2 nm (PdI 0.178) in cyclohexane was determined, while by TEM, the diameter of the bare particles was determined as (8.1 ± 1.1) nm. As shown in Electronic Supplementary Material (ESM) Fig. S2, the particles are of uniform cubic shape and monodisperse.

**Fig. 4:**
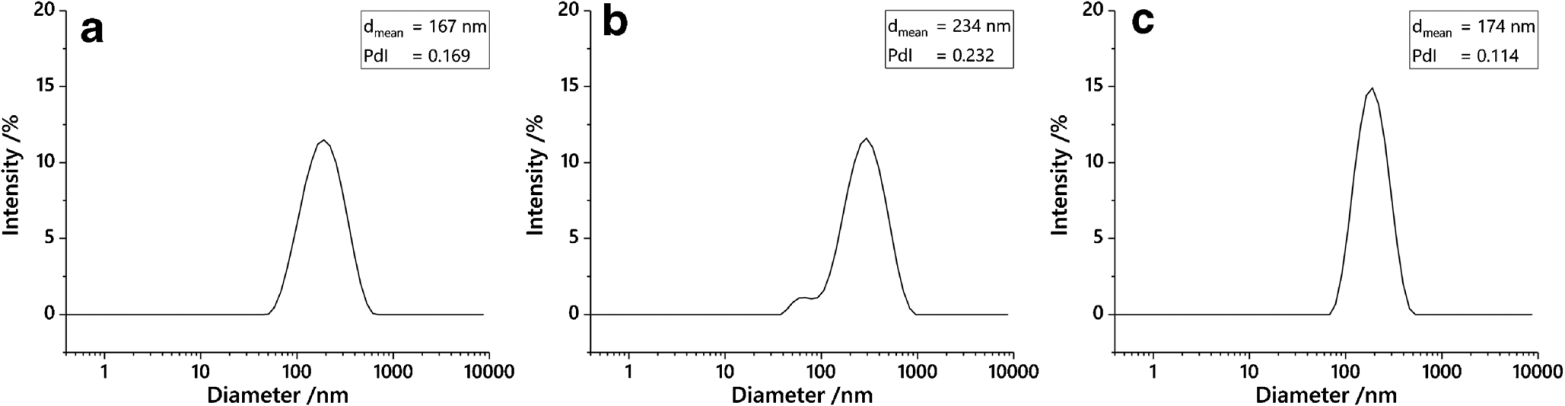
DLS measurement of original b-liposomes (**a**), optimized b-liposomes (**b**), and i-liposomes (**c**). Optimized b-liposomes are improved regarding MNP entrapment and performance in the assay, not regarding their size distribution. Due to only one extrusion step, the size distribution is broader than with two consecutive steps

Liposomes were characterized according to their hydrodynamic diameter determined by DLS measurements, which is around 170 nm for non-optimized (original) liposomes extruded through 1.0 and 0.4 μm membranes, and 234 nm for optimized liposomes with only one extrusion step at 1 μm pore size (Fig. 4). The zeta potential of all liposomes is ~ − 20 mV, which is characteristic for liposomes with this lipid composition and typically leads to high colloidal stability (for more information see ESM). TEM images show a Janus-shaped incorporation of particles in the bilayer as also described by other researchers previously [16] (see ESM Fig. S2).

**Fig. 5:**
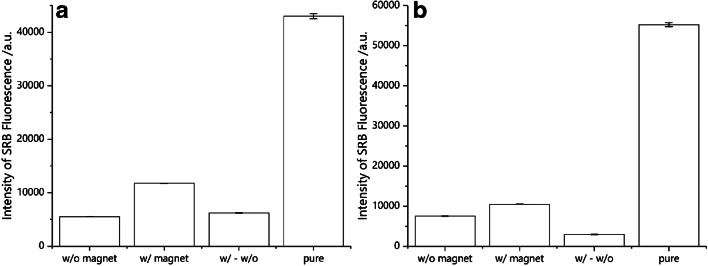
Encapsulation efficiency of optimized b-liposomes (**a**) and i-liposomes (**b**) as determined by magnetic separation and fluorescence measurements (*n* = 4)

As the liposome mixture is composed of magnetically modified liposomes as well as liposomes without incorporated particles, this fraction was determined by measuring the fluorescence signal with and without magnetic separation and comparing them (Fig. 5). Therefore, liposomes were washed twice in the absence (w/o magnet) or presence (w/ magnet) of a magnetic field, respectively, and the resulting intensity values were subtracted (w/ - w/o) to eliminate non-specific binding effects. The value was then compared with unwashed liposomes with the same starting concentration (pure). For optimized liposomes with MNPs incorporated into the lipid bilayer (b-liposomes), it was found that (14.5 ± 0.2)% of the liposomes were magnetic in the original solution, while for encapsulating MNPs in the hydrophilic inner cavity of the liposomes together with marker molecules (i-liposomes), only a fraction of (5.35 ± 0.9)% was determined to be magnetic (Fig. 5).

An explanation for this phenomenon is on the one hand the large hydrodynamic diameter of these particles, which prevents incorporation of theoretical possible high particle quantities per liposome, and on the other hand, the lower amount of particles used for i-liposomes due to the higher cost and lower concentration of commercial particle solutions. Based on theoretical calculations, a maximum of ~ 125 MNPs (spherical, 30 nm diameter) could fit inside a liposome (spherical, 170 nm inner diameter), if fully packed (for calculations see ESM). Nevertheless, this is a purely theoretical number as MNP solutions can hardly be prepared that concentrated and as the hydrodynamic diameter of particles and their electrostatic repulsion prevent close packing. In addition, achieving a theoretical amount of one particle per liposome would require commercial particles for US$350 per liposome batch. Therefore, a particle concentration was chosen that would theoretically equip 16% of the liposomes with magnetic properties. Actually, 5% of liposomes were found to be magnetic.

In contrast, the MNP concentration chosen for bilayer incorporation synthesis was supposed to equip more or less 100% of liposomes with magnetic features (56 particles per liposome), but only a 15% efficiency was detectable. This indicates that the bilayer entrapment is sterically and thermodynamically more challenging. Nevertheless, the costs for the production of the here required amount of magnetic nanoparticles for insertion into the bilayer is around US$10 per liposome batch and can even be lowered when scaling the particle production up (for calculations see ESM).

### Development of optimized bilayer insertion liposomes

To validate the magnetic abilities of the synthesized magnetosomes, a DNA hybridization sandwich assay was performed in parallel with and without the presence of an external magnetic field. It should be pointed out that in such assays, more than hundreds of liposomes will contribute to signals recorded. A natural distribution in liposome size and magnetization therefore only minimally impacts the assay performance as can be seen from all standard deviations observed. In a first attempt with b-liposomes (ESM Fig. S3), limits of detection (LOD) and quantification (LOQ) (zero value plus three and ten times standard deviation, respectively) and the maximum signal-to-noise ratio (max S/N) got surprisingly poorer when applying a magnetic field (1.1- to 1.2-fold higher without than with magnet, see Table 1). We assume that this was due to increased non-specific binding of the b-liposomes occurring. Therefore, optimization of these liposomes was necessary.

Three synthesis parameters were investigated regarding possible optimization of the system: the cholesterol fraction of the total lipid composition, the pore size of employed extrusion membranes, and the overall synthesis method.

**Fig. 6:**
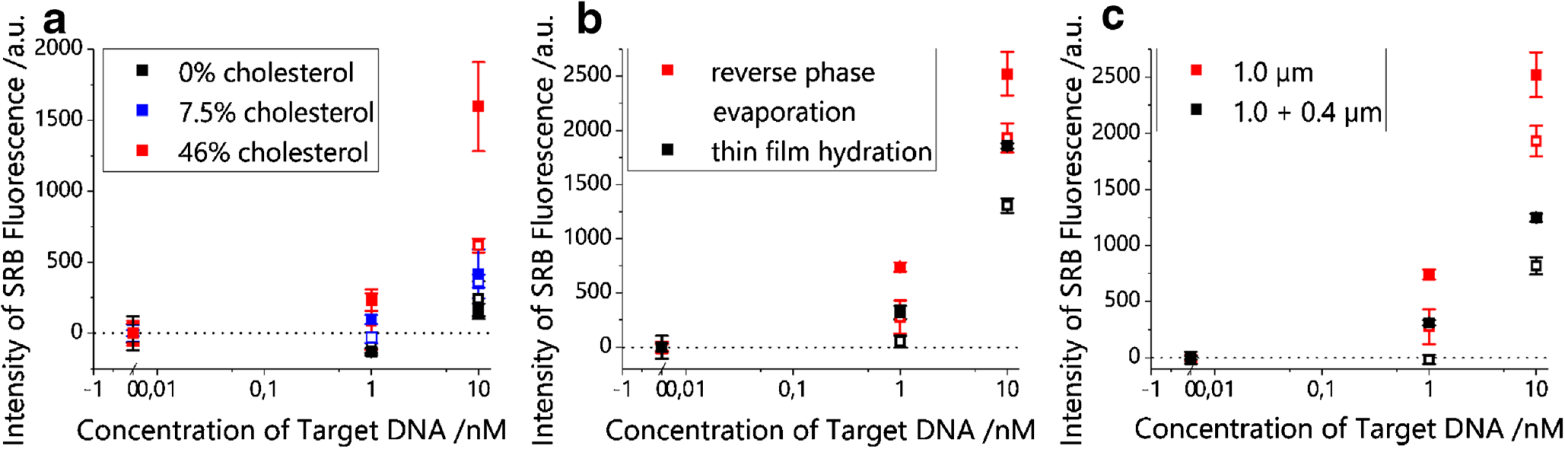
DNA hybridization assays performed for different b-liposome systems once in the absence (empty squares) and once in the presence (filled squares) of an external magnetic field. Cholesterol content (**a**), synthesis method (**b**), and extrusion steps (**c**) were varied (*n* = 3)

Cholesterol is added to the lipid composition to stabilize the membrane, as it reduces on the one hand the repulsion of charged headgroups by increasing the headgroup spacing, and on the other hand, the motion of hydrocarbon chains by increased Van der Waals interactions [20]. But this effect also stiffens the membrane, which likely hinders the intercalation of nanoparticles between the two rigid bilayer sheets as the membrane has to be arranged in a more distorted structure. Therefore, reduction of the cholesterol content was investigated to yield higher membrane fluidity and thus higher MNP encapsulation. Unfortunately, as shown in Fig. 6a, a reduction of the cholesterol content did not improve the assay. Specifically, with 7.5% cholesterol, the assay in the presence of a magnetic field still yields higher signals than without magnetic field. However, the results are overall worse than with 46% cholesterol. When no cholesterol is included in the lipid mixture, the results are exactly opposite to those obtained with 7.5% cholesterol. A possible explanation for this phenomenon is that, without a stabilizing cholesterol present, lipid-coated nanoparticles form, which cover the surface of the microtiter plate and hinder binding of dye-filled liposomes to the target DNA. This would also explain why some data points show lower fluorescence than the blank solutions. In the presence of target DNA, the coated particles can bind and shield the plate surface against unspecific binding of dye-filled liposomes, which lowers the background signal, while without target DNA in the blank samples, some of the particles can be removed by shaking and washing and liposomes can adsorb.

Secondly, thin film rehydration was investigated and compared with reverse phase evaporation, assuming that the pre-formation of lipid films will favor MNP integration into the lipid bilayer. Unfortunately, no improvement of the assay could be achieved by changing the synthesis method (Fig. 6b).

Finally, the pore size of the applied extrusion membranes was investigated. Extrusion is performed to yield uniform unilamellar vesicles with a narrow size distribution. These are more stable than a liposome dispersion with a broad size distribution, as those can fuse or grow by Ostwald ripening and get unstable due to an unfavorable surface curvature [21]. Also, uniform liposomes are preferred in analytical assays to improve the reproducibility of binding events. However, a visibly high loss of magnetic particles was observed during the extrusion process, when liposomes are ripped apart to form in a uniform and unilamellar way again at the other side of the membrane. Therefore, it was investigated if the use of only one extrusion through a membrane with 1.0 μm pores instead of two consecutive extrusions through membranes with 1.0 and 0.4 μm pores would yield better results, finding a compromise between homogenizing the liposome size and severe particle loss. In fact, it was found that through reducing the number of extrusion steps, higher signals and a steeper slope of the signal curve could be achieved (Fig. 6c). The liposome diameter went from 170 to 230 nm (PdI 0.11 to 0.23), which remains in the stable unilamellar size range for these bioanalytical liposomes [22].

### Comparison of magnetic liposome systems

**Fig. 7:**
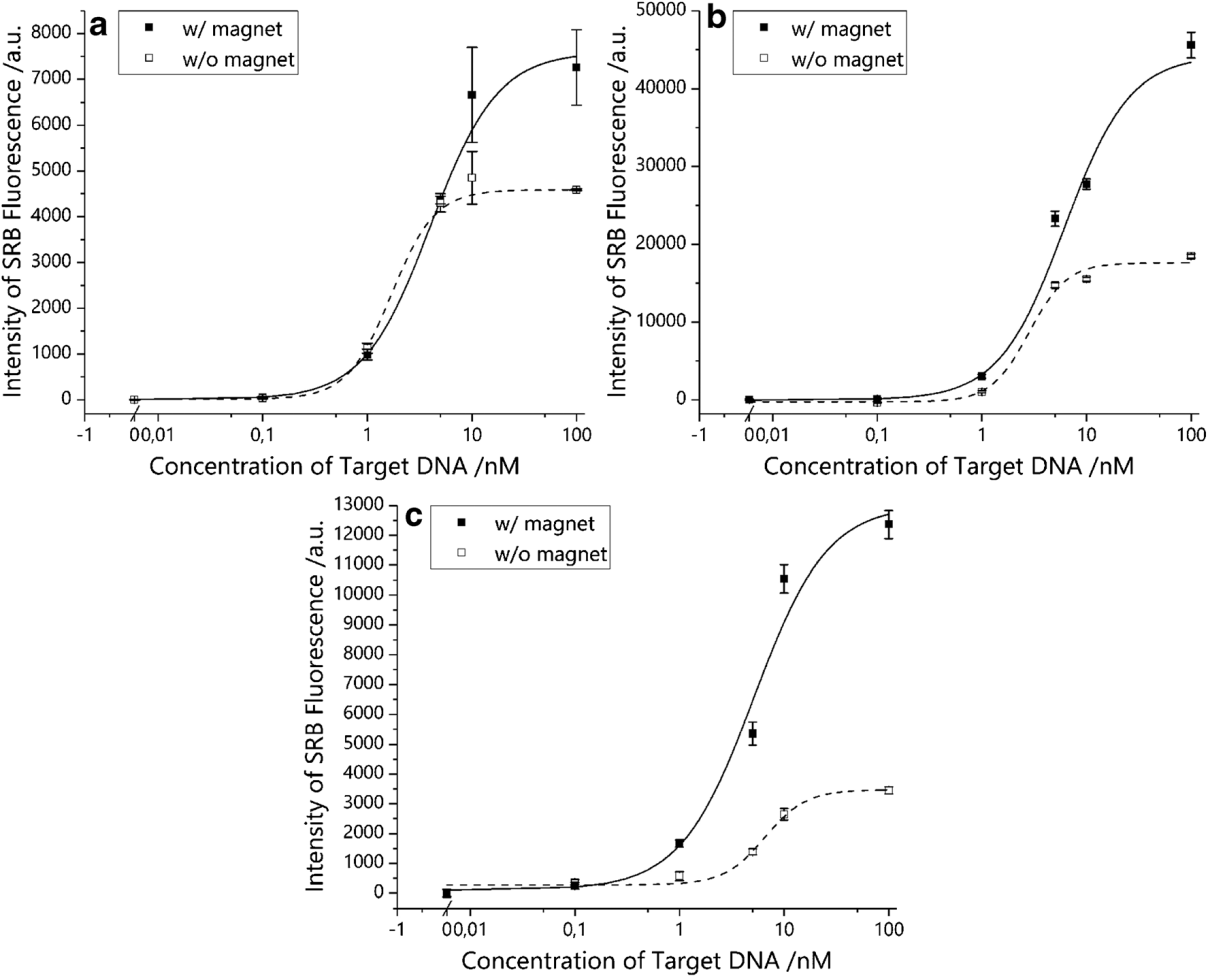
DNA sandwich hybridization assay with optimized b-liposomes before (**a**) and after (**b**) magnetic separation and concentration adjustment to 14.5%, with the same is shown for i-liposomes after magnetic separation and concentration adjustment to 5% (**c**) (*n* = 3). For the graph for i-liposomes without magnetic separation, please refer to ESM Fig. S3 right

For an optimized b-liposome system, synthesis by reverse phase evaporation, a cholesterol content of 45% and only one extrusion step with a pore size of 1.0 μm were chosen. Performance investigation by DNA hybridization revealed very low LOD and LOQ, and a high improvement with the use of an external magnetic field could be achieved (~ 3-fold reduction of LOD/LOQ and increase of max S/N, respectively, see Table 1). Interestingly, after magnetic separation and concentration adjustment to 14.5%, improvement factors were in the same range as prior magnetic separation, while the LOD and LOQ rose significantly (Fig. 7).

**Table 1:** Statistics for magnetosome systems. Improvement for LOD and LOQ (the lower the better) is defined as *without magnet* divided by *with*, for sensitivity and maximum signal to noise ratio (the higher the better) as *with* divided by *without*

		LOD/pM	LOQ/pM	Sensitivity/slope^1^	Max S/N^2^
Original b-liposomes	W/ magnet	449	1300	228	48
	W/o magnet	386	1070	142	55
	Improvement	0.9	0.8	1.6	0.9
Optimized b-liposomes	W/ magnet	42	102	5675	1404
	W/o magnet	141	253	5251	527
	Improvement	3.4	2.5	1.1	2.7
Optimized b-liposomes after magnetic separation	W/ magnet	162	388	34,171	491
	W/o magnet	525	885	23,300	176
	Improvement	3.2	2.3	1.5	2.8
Original i-liposomes	W/ magnet	137	257	5966	813
	W/o magnet	97	205	5043	615
	Improvement	0.7	0.8	1.2	1.3
i-Liposomes after magnetic separation	W/ magnet	314	880	8696	95
	W/o magnet	2459	4899	3843	31
	Improvement	7.8	5.6	2.3	3.0

^1^a.u.·log_10_nM^−1^

^2^Maximum signal to noise ratio

In the case of i-liposomes, an assay showed no difference with or without applied external magnetic field (ESM Fig. S3), yet for the separated magnetic fraction, significant improvement for LOD, LOQ, sensitivity, as well as max S/N could be achieved, although LOD and LOQ again rose after the removal of non-magnetic liposomes.

**Fig. 8:**
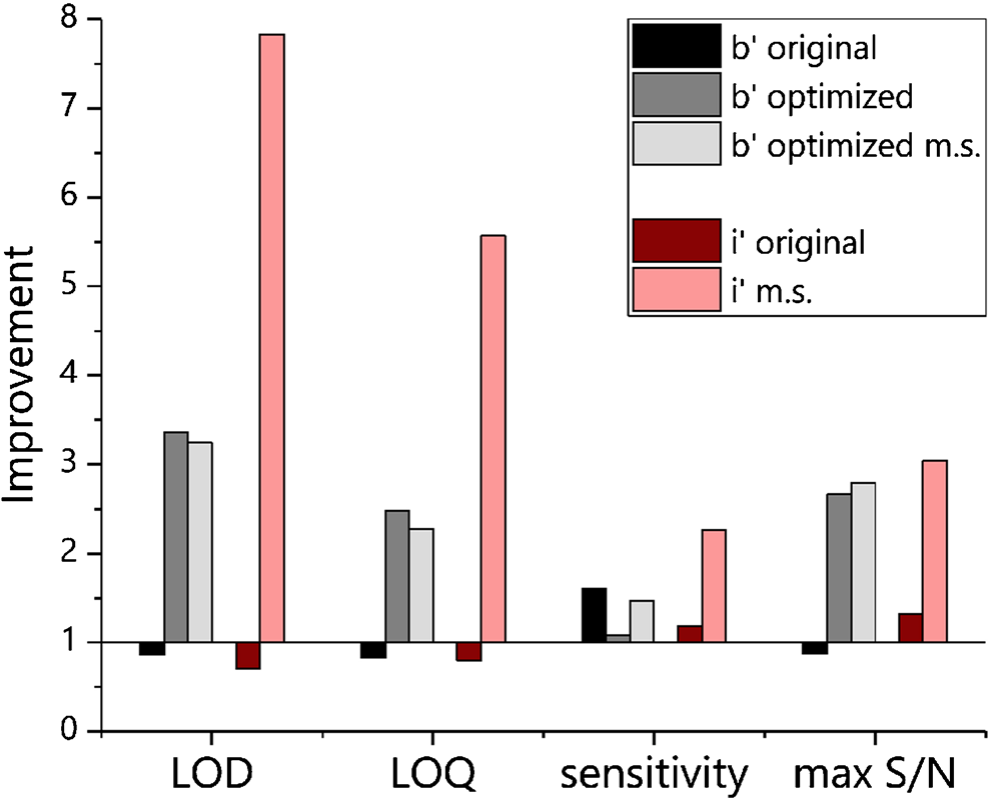
Comparison of improvement factors of different magnetosome systems and their optimizations (= 1: no difference between with and without magnet; > 1: improvement with magnet; < 1: worsening with magnet). *Gray*: b-liposomes (b′), original, optimized, and optimized after magnetic separation (m.s.) with concentration adjustment to 14.5%. *Red*: i-liposomes (i′), original and after magnetic separation with concentration adjustment to 5%. LOD and LOQ are improved when the value is lower with magnet than without magnet; sensitivity and signal to noise ratio are improved if the value is rising by application of a magnetic field

When comparing the different magnetosome systems (Table 1, Fig. 8), it can be observed that in total, the lowest LOD was obtained with optimized b-liposomes at 42 pM, being 3.4 times lower than in the absence of a magnetic field, while i-liposomes showed generally higher LODs, probably due to the lower fraction of liposomes with magnetic features in the mixture (only around 5%). Separating out non-magnetic liposomes in all cases increased the difference between the results for the absence and presence of a magnetic field, yielding much better values with magnet, but the overall LODs worsened. We assume that, without magnetic separation, the non-magnetic liposomes present in the solution are dragged towards the magnetic field by their magnetic counter parts and thus higher binding rates can be observed. In contrast, when the same is done after magnetic purification, this additional contribution ceases. Here, i-liposomes showed higher improvement than b-liposomes, possibly due to more room for progress as only a twentieth of the liposomes are magnetic instead of a seventh for b-liposomes.

## Conclusion

In previous studies, the proof of principle of b-liposome generation and the achievement of an overall improvement factor of 15-fold in a DNA assay have been shown [15]. These liposomes were larger (hydrodynamic diameter of 357 nm) than those investigated here and not further studied with respect to the percentage of magnetization. Here, we carefully studied two possible strategies to magnetize liposomes while keeping their signal amplification capability intact. Optimization strategies were investigated and liposomes were characterized in detail by DLS, TEM, ICP-OES, and fluorescence measurements.

At first glance, it was found that the inclusion of magnetic particles in the lipid bilayer yielded a higher efficiency than their encapsulation within the inner cavity. In fact, with the concentration chosen, 16% of liposomes are supposed to be magnetic, while our results found 5% magnetic liposomes, equaling a 30% overall efficiency. In contrast, the bilayer incorporation synthesis was significantly less efficient. While a more or less 100% magnetization of liposomes was to be expected based on the MNP concentration chosen, only a 15% efficiency was detectable. This suggests that, with the concentrations chosen, the inner cavity encapsulation strategy is two times more efficient than the bilayer entrapment, which we assume is caused by the fact that the bilayer entrapment is sterically and thermodynamically more challenging.

Nevertheless, the costs for the production of the required amount of magnetic nanoparticles for insertion of one particle per liposome into the bilayer is less than US$1 per liposome batch and can even be lowered when scaling the synthesis up. In contrast, achieving this amount by inner cavity entrapment would require about US$350 per liposome batch. In the future, we will overcome this challenge by coating our own MNPs with stable hydrophilic ligands and hence find strategies for low-cost hydrophilic MNPs. We can then also investigate easily MNPs with different size and composition, such as those made of cobalt, to achieve higher magnetization. Ultimately, it will be interesting to develop strategies of combined immunomagnetic separation and magnetically enhanced detection in just one assay.

## Electronic supplementary material

ESM 1 (PDF 1.10 mb)
