# Supplementary material for: Magnetosomes for bioassays by merging fluorescent liposomes and magnetic nanoparticles: encapsulation and bilayer insertion strategies
Source: Anal Bioanal Chem. 2020 Feb 18;412(24):6295–305. doi: 10.1007/s00216-020-02503-0 (PMC7442762; doi:10.1007/s00216-020-02503-0)
Supplement: Supplementary file 1 — (PDF 1.10 mb) [file 216_2020_2503_MOESM1_ESM.pdf]

Magnetosomes for bioassays by merging fluorescent liposomes and magnetic nanoparticles: Encapsulation and bilayer insertion strategies

Cornelia A. Hermann[1], Carola Hofmann[1], Axel Duerkop[1], Antje J. Baeumner[1,*]

[1] Institute for Analytical Chemistry, Chemo- and Biosensors, University of Regensburg, Universitätsstraße 31, 93053 Regensburg, Germany

Supporting Information

1. Characterization

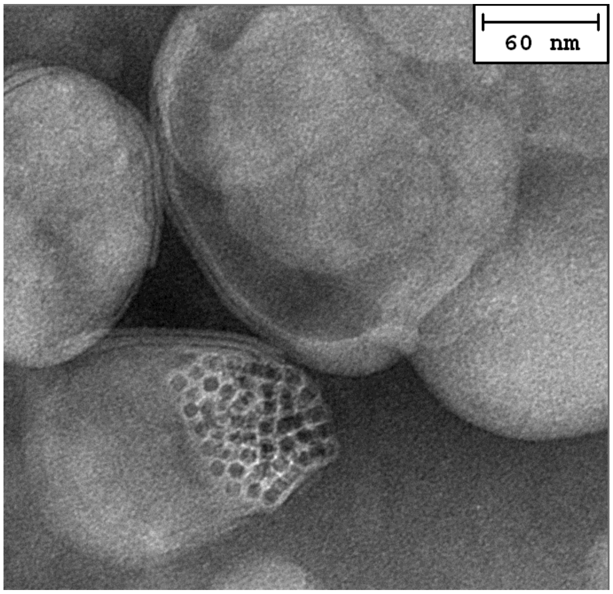

Fig. S1 TEM image of original b-liposomes with incorporated MNPs with hydrophobic surface coating. To reduce the distortion of the membrane, particles accumulate at one side instead of spreading across the whole bilayer, forming Janus shaped vesicles as also reported by other scientists previously [1].

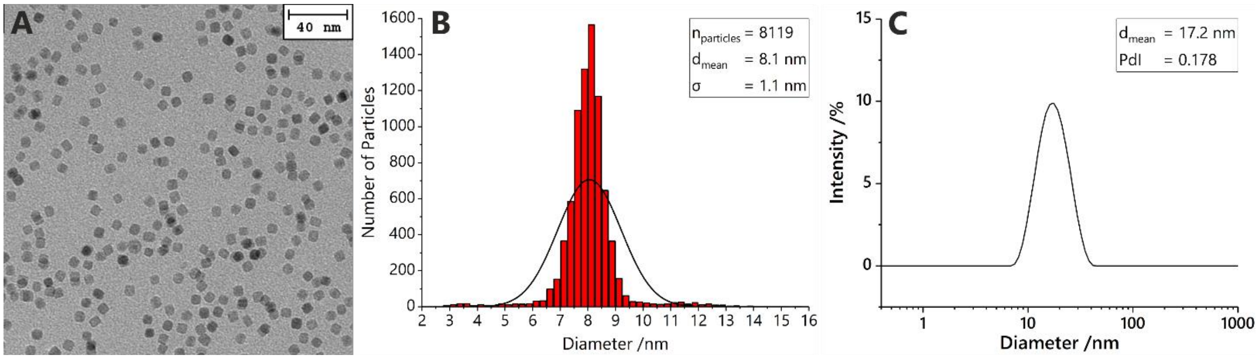

Fig. S2 TEM image (A), particle size distribution as obtained from TEM images (B) and DLS (C) of MNPs. The particles possess an average diameter d_{mean} of 8.1 nm with a standard deviation σ of 1.1 nm and a solvodynamic diameter d_{mean} of 17.2 nm with a PdI of 0.178 obtained in cyclohexane.

Table S1 Hydrodynamic diameter, PdI and zeta potential of compared liposome systems

	hydrodynamic diameter /nm (PdI)	zeta potential /mV
b-liposomes original	167 (0.169)	(-20.6±1.6)
b-liposomes optimized	234 (0.232)	(-20.5±1.0)
i-liposomes original	174 (0.114)	(-21.3±1.4)

2. Calculation of Particle Amount per Liposome and Costs for Particles

The theoretical number of particles that fit into the inner cavity per liposome N_{tic} is calculated according to Equation 1:

$$N_{tic} = \frac{inner\ volume\ of\ one\ liposome}{volume\ of\ one\ particle} \cdot PD = \left(\frac{d_L - (2 \cdot d_b)}{d_p}\right)^3 \cdot PD = \left(\frac{174\ nm - 2 \cdot 4\ nm}{30\ nm}\right) \cdot 74\% \quad (1)$$
$$= 125\ particles$$

where d_L is the outer liposome diameter, d_b is the bilayer thickness [2], d_p is the particle diameter and PD is the packaging density [34].

The actual number of particles in the inner cavity per liposome N_{aic} is calculated as follows (Equation 2):

$$N_{aic} = \frac{number\ of\ particles}{number\ of\ liposomes} = \frac{\frac{total\ volume\ of\ particles}{volume\ of\ one\ particle}}{\frac{number\ of\ lipids}{number\ of\ lipids\ per\ liposome}} = \frac{\frac{m_P}{\rho \cdot \frac{\pi}{6} \cdot d_P}}{\frac{n_L \cdot N_A \cdot A_{mean}}{\pi \cdot \left(d_L{}^2 + \left(d_L - (2 \cdot d_b)\right)^2\right)}}$$
$$= \frac{\frac{1\ mg}{5.18\ \frac{g}{cm^3} \cdot \frac{\pi}{6} \cdot 30\ nm}}{\frac{60\ \mu mol \cdot N_A \cdot 0.42\ nm^2}{\pi \cdot ((174\ nm)^2 + (174\ nm - 2 \cdot 4\ nm)^2)}} = 0.16\ particles \qquad (2)$$

where m_P is the inserted mass of particles, ρ is the density of iron(III)oxide [4], n_L is the inserted amount of lipids, N_A is Avogadro's constant and A_{mean} is the mean area per lipid molecule [5].

For the calculation of the actual number of particles in the bilayer per liposome N_{ab} (based on the inserted amount of particles), Equation 2 was adapted, as these particles are of cubic shape instead of spherical shape as in the case of inner cavity encapsulation (Equation 3):

$$N_{ab} = \frac{\frac{m_P}{\rho \cdot \left(\frac{d_P}{\sqrt{2}}\right)^3}}{\frac{n_L \cdot N_A \cdot A_{mean}}{\pi \cdot \left(d_L{}^2 + \left(d_L - (2 \cdot d_b)\right)^2\right)}} = \frac{\frac{1\ mg}{5.18\ \frac{g}{cm^3} \cdot \left(\frac{30\ nm}{\sqrt{2}}\right)^3}}{\frac{60\ \mu mol \cdot N_A \cdot 0.42\ nm^2}{\pi \cdot ((174\ nm)^2 + (174\ nm - 2 \cdot 4\ nm)^2)}} = 56\ particles \qquad (3)$$

The theoretical mass of particles that has to be inserted in the synthesis to achieve one particle per liposomes $m_{1P/L}$ (*ic* for inner cavity and *b* for bilayer) was calculated according to Equation 4 and 5, respectively:

$$m_{1P/L\ ic} = \rho \cdot volume\ of\ one\ particle \cdot number\ of\ liposomes$$
$$= 5.18\ \frac{g}{cm^3} \cdot \frac{\pi}{6} \cdot 30\ nm \cdot \frac{60\ \mu mol \cdot N_A \cdot 0.42\ nm^2}{\pi \cdot ((174\ nm)^2 + (174\ nm - 2 \cdot 4\ nm)^2)} = 6\ mg \qquad (4)$$

$$m_{1P/L\ b} = 5.18\ \frac{g}{cm^3} \cdot \left(\frac{30\ nm}{\sqrt{2}}\right)^3 \cdot \frac{60\ \mu mol \cdot N_A \cdot 0.42\ nm^2}{\pi \cdot ((174\ nm)^2 + (174\ nm - 2 \cdot 4\ nm)^2)} = 0.04\ mg \qquad (5)$$

With this amounts and masses, also the costs for each synthesis were calculated, either by simply multiplying the amount of particles for inner cavity encapsulation with the price of these particles, or - for the self-synthesized particles for bilayer insertion - taking into account the prices of the chemicals and the working time per synthesis multiplied with an average salary of a laboratory assistant in Germany. Of course, these calculations are just rough approximations and make no claim to completeness, but still they show the enormous discrepancy between both methods.

3. DNA Hybridization Assay Preliminary Studies

To validate the magnetic abilities of the synthesized magnetosomes, a DNA hybridization sandwich assay was performed in parallel with and without the presence of an external magnetic field (Fig. S3 left). In a first assay with original b-liposomes, LOD, LOQ and the maximum signal to noise ratio (max S/N) were 1.2 times better without than with magnet, respectively, although the sensitivity increased by 1.6 times. Therefore, optimization of this assay was necessary.

Another liposome system encapsulated hydrophilic magnetic particles inside the inner cavity of the liposomes, aside with the signal molecules. A first assay showed no significant change when conducting the assay with or without the presence of an external magnetic field (Fig. S3 right).

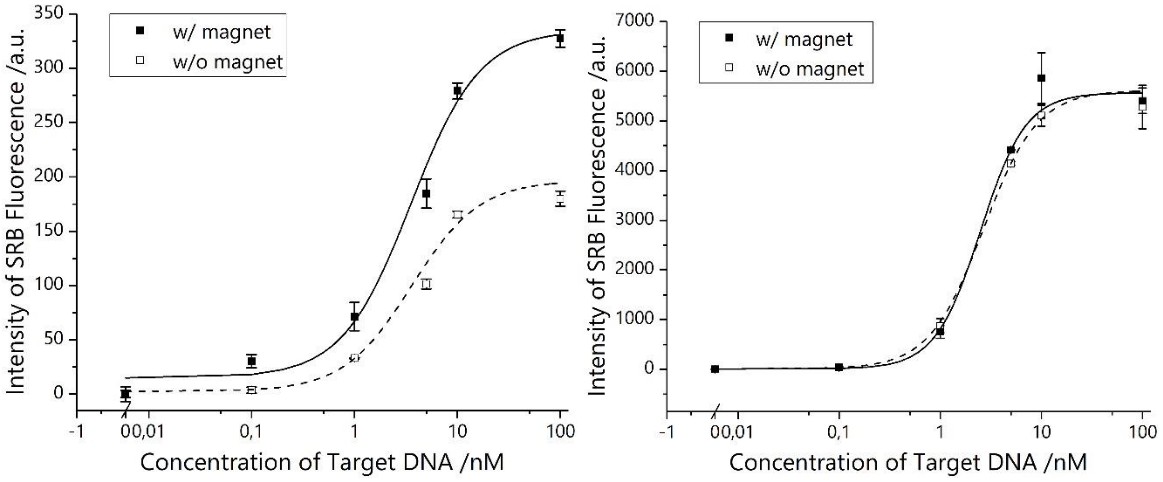

Fig. S3 DNA hybridization sandwich assay with original b-liposomes (left) and original i-liposomes (right), performed with and without the presence of an external magnetic field. (N=3)

Error Bars were calculated by the following procedure: For each concentration, three or four wells (see number N in figure caption) were pipetted in parallel to determine the pipetting error. Then each microtiterplates was measured three consecutive times to determine the error of the instrument. Gaussian propagation of uncertainty was used to calculate the error bars from these two values.

4. Liposome Stability

For examination of the long-term stability of i- and b-liposomes, the size distribution of liposomes was determined with dynamic light scattering directly after synthesis as well as after 9 and 11 months of storage (4 °C in the dark), respectively. Figure S4 shows the distribution of the hydrodynamic diameter for two different batches of b-liposomes and one batch of i-liposomes. No significant change in hydrodynamic diameter could be observed and the polydispersity index is still very low. Thus, and as no visible precipitation

or lysis of the liposomes is observable, it can be assumed that magnetic liposomes are stable over at least 9 months of storage.

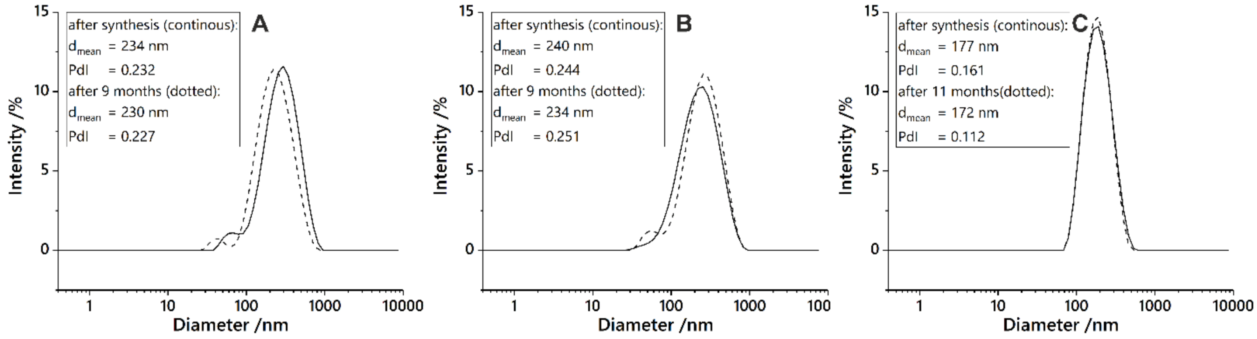

Fig. S4 Size distribution of liposomes determined by dynamic light scattering directly after synthesis and after 9 and 11 months of storage, respectively. A/B: Two different batches of b-liposomes, C: i-liposomes.

For examination of the stability of liposomes under electromagnetic attraction, the size distribution of liposomes was determined with dynamic light scattering, then the cuvettes with liposome solution were placed next to a strong neodymium magnet for 60 min and after this time, the size distribution was measured again. As visible in Figure S5, no significant change in diameter and distribution was observable, as well as no visible precipitation or lysis. Thus, liposomes are assumed stable under the influence of an external magnetic field.

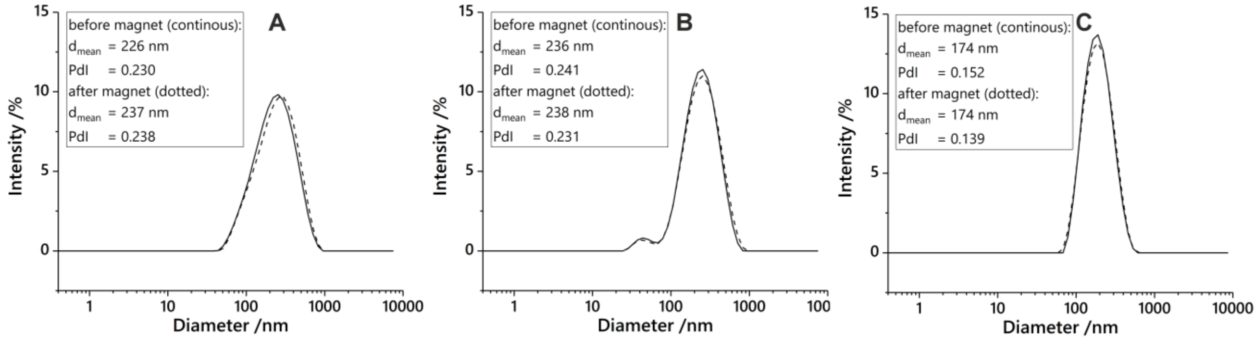

Fig. S5 Size distribution of liposomes determined by dynamic light scattering before and after application of an external magnetic field for 60 min. A/B: Two different batches of b-liposomes, C: i-liposomes.

5. Experiment Flow

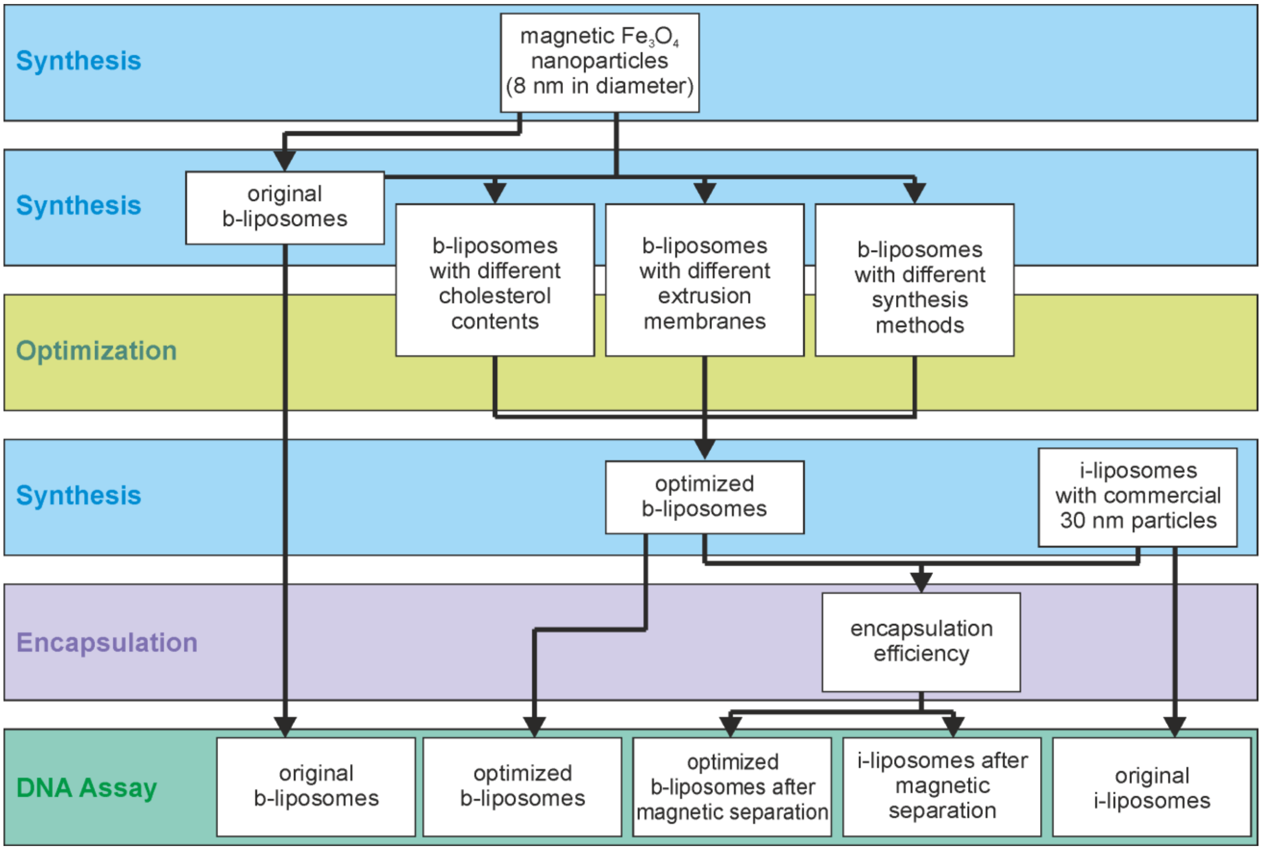

Fig. S6 Flow chart illustrating the experiments performed for this publication.

References:

1. Bonnaud C, Monnier CA, Demurtas D, Jud C, Vanhecke D, Montet X, Hovius R, Lattuada M, Rothen-Rutishauser B, Petri-Fink A (2014) Insertion of nanoparticle clusters into vesicle bilayers. ACS nano 8(4):3451–3460

2. Israelachvili JN, Mitchell DJ (1975) A model for the packing of lipids in bilayer membranes. Biochim. Biophys. Acta(389):13–19

3. Hales TC (2005) A proof of the Kepler conjecture. Annals of Mathematics(162):1065–1185

4. Schwertmann U, Cornell RM (2008) Iron oxides in the laboratory: preparation and characterization. John Wiley & Sons

5. Fenzl C, Genslein C, Domonkos C, Edwards KA, Hirsch T, Baeumner AJ (2016) Investigating non-specific binding to chemically engineered sensor surfaces using liposomes as models. The Analyst 141(18):5265–5273
